# Innate immunity and carbohydrate metabolism alterations precede occurrence of subclinical mastitis in transition dairy cows

**DOI:** 10.1186/s40781-015-0079-8

**Published:** 2015-12-23

**Authors:** Elda Dervishi, Guanshi Zhang, Dagnachew Hailemariam, Suzana M. Dunn, Burim N. Ametaj

**Affiliations:** Department of Agricultural Food, and Nutritional Science, University of Alberta, Edmonton, AB T6G 2P5 Canada

**Keywords:** Subclinical mastitis, Dairy cows, Transition period, Blood metabolites, Acute phase proteins, Cytokines

## Abstract

**Background:**

This study examined whether activation of innate immunity and alterations of carbohydrate and lipid metabolism precede development of subclinical mastitis (SCM).

**Methods:**

Blood samples were collected from the coccygeal vein from 100 Holstein dairy cows at -8, -4, disease diagnosis week, and +4 weeks postpartum. Six healthy cows (controls – CON) and six cows that showed clinical signs of SCM were selected for serum analyses. All serum samples were analyzed for acute phase proteins (APP) haptoglobin (Hp) and serum amyloid A (SAA); proinflammatory cytokines including interleukin 1 (IL-1), IL-6, and tumor necrosis factor (TNF) and serum lactate, BHBA, and NEFA concentration. Data of DMI, milk production, and milk composition were recorded and analyzed.

**Results:**

The results showed that cows with SCM had greater concentrations of SAA, TNF (*P* < 0.01), and lactate before expected day of parturition (*P* < 0.05) compared to CON cows. Cows with SCM showed greater concentrations of lactate starting at -8 weeks (*P* < 0.05) and TNF starting at -4 weeks prior to the expected day of parturition (*P* < 0.01). Interestingly, at -4 weeks, concentrations of IL-1 and Hp were lower in cows with SCM compared to healthy cows (*P* < 0.01) followed by an increase during the week of disease diagnosis (*P* < 0.05). Subclinical mastitis was associated with lower DMI, at -4 weeks before calving, milk production (*P* < 0.05) and increased somatic cell counts (SCC) (*P* < 0.01).

**Conclusions:**

Results of this study suggest that SCM is preceded by activated innate immunity and altered carbohydrate metabolism in transition dairy cows. Moreover the results support the idea that Hp, lactate, and SAA, at -8 weeks, and TNF and IL-1 at -4 weeks can be used as early indicators to screen cows during dry off for disease state.

## Background

Subclinical mastitis (SCM) is the main form of mastitis in dairy cattle and its prevalence ranges between 20 and 50 % of the cows in a herd [1, 2]. During SCM there are no visible alterations in the appearance of milk or udder; however, milk production decreases, somatic cell count increase (SCC), bacterial pathogens are present in the milk, and the milk composition is altered [3]. In Canada the regulatory limit of SCC is 400,000 cells/mL of raw milk [4]; however, International Dairy Federation (1997) has determined that SCC > 200.000 cells/mL of milk suggests presence of mastitis. A somatic cell score limit of less than 100,000 cells/mL is suggested to be a healthy level of SCC [5]. Moreover, the SCC for the composite milk of a cow should not exceed 100,000 cells/mL [6]. Subclinical mastitis can cause major economic losses due to lowering of milk production and lowering of shelf life of milk. The cost of 1 case of SCM has been estimated in the range of $200/year [7].

The inflammatory and immune responses to mastitis infection affect the animal welfare and wellbeing and are associated with an important decrease in milk production and lower reproductive performance [8, 9]. The negative consequences of mastitis on the reproductive efficiency of dairy cows are not limited to its clinical form but are also observed when the disease is at its subclinical stage [10]. Early identification of udder health problems is essential for dairy industry to ensure the animal well-being and also the milk quality and productivity. Moreover, cows with SCM should be considered as a risk for spreading mastitis pathogens within and between herds and as such are of important concern [11].

Several studies have reported alterations in blood and milk metabolites, proinflammatory cytokines, and acute phase proteins in cows with SCM. For example, increases in the concentration of lactate in the milk of cows during mastitis, suggest that lactate could be a good indicator of udder health [12, 13]. In addition, the expression of interleukin (IL)-6 has been reported to increase in cows with SCM [14]. It is believed that IL-1 and IL-6 are the two main triggers for production of acute phase proteins (APP) by liver hepatocytes [15, 16]. Several APP have been well investigated as biomarkers of mastitis in cows [16–18]. For example, concentrations of serum amyloid A (SAA) are strongly elevated during mild, moderate, and clinical mastitis [19–21]. In other studies it was reported that concentration of haptoglobin (Hp) in the serum increases in cows affected by clinical or subclinical mastitis [22–25]. These findings suggest that both Hp and SAA might be potential screening biomarkers for bovine mastitis.

An increasing body of evidence suggests that changes in the metabolic and innate immune variables in the systemic circulation might start in the early stages of disease. Therefore early diagnosis or detection of those alterations before development of subclinical disease could help in developing preventive strategies in the future. Therefore the objectives of the present investigation were to search for early screening biomarkers of disease state in the blood of transition dairy cows starting at -8 and -4 weeks prior to the expected day of parturition, during the week of disease diagnosis, and up to +4 weeks postpartum. The postpartum purpose of the monitoring is to evaluate how long the disease lasts after initiation. A group of variables traditionally related to innate immunity and carbohydrate and lipid metabolism will be included in the testing evaluation.

## Methods

### Animals and diets

All experimental procedures were approved by the University of Alberta Animal Policy and Welfare Committee for Livestock and animals were cared for in accordance with the guidelines of the Canadian Council on Animal Care [26].

One hundred pregnant Holstein dairy cows at the Dairy Research and Technology Centre, University of Alberta (Edmonton, AB, Canada), were used in a longitudinal study. Six pregnant Holstein dairy cows (average parity: 3.1 ± 0.4) were diagnosed with subclinical mastitis and six healthy control cows (CON) that were similar in parity, (average parity: 3.2 ± 0.3), age, and body condition score (BCS), were selected for this nested case-control study.

The experimental period lasted for 13 weeks starting from -8 weeks before parturition to +4 weeks postpartum (i.e. -8 weeks to +4 weeks, 0 week means the week of calving) for each cow. Cows were housed in individual tie stalls, bedded with sawdust and with free access to water throughout the experiment. Shortly before calving cows were transferred to the maternity barn and returned to their stalls on the following day of parturition. Diets were offered as TMR for ad libitum intake once daily at 08.00 h to allow approximately 5 % orts. All TMR were formulated to meet or exceed the nutrient requirements of dry and early 680 kg lactating cows as per National Research Council guidelines. Individual dry matter intake (DMI) was recorded daily throughout the 13 weeks period. Since the onset day of lactation, cows were milked in their stalls twice per day at 05.00 and 16.00 h, and individual milk yield (MY) was recorded electronically. Milk composition like crude protein (CP), milk fat, lactose, somatic cell count, milk urea nitrogen (MUN), and total solids (TS) were analyzed by mid-infrared spectroscopy (MilkoScan 605; A/S Foss Electric, Hillerød, Denmark) at the DHI Central Milk Testing Laboratory in Edmonton, Alberta. Milk composition was analyzed at +2, +3, +5, and +7 weeks post-partum.

### Monitoring of clinical health status

Overall health status (HS) of cows was monitored daily, based on clinical signs of disease by trained individuals and on a weekly basis by a veterinary practitioner. All periparturient diseases and veterinary treatments were recorded for each cow throughout the entire experimental period. Based on the artificial insemination (AI) data, supported with the information of pregnancy diagnosis, the expected date of parturition was fixed by adding 280 days from the day of AI. All cows were monitored daily starting at -8 weeks prior to the expected date of calving and continuing up to +8 weeks postpartum. The various external symptoms observed were gait, general appearance, appetite, alertness, rectal temperature, ease of calving, body condition score, body temperature, vaginal discharges (color and consistency), udder edema, flakes in the milk, and pain in the legs.

In this study, SCM was diagnosed according to the farm standard operating procedures. A subclinical mastitis case was diagnosed if the cow had an elevated SCC but milk appears normal. Value of SCC > 200,000 for two or more consecutive reports were used for confirmation of SCM. No treatment was given to the animals unless indicated by a veterinarian.

### Sample collection

Blood samples were obtained from the coccygeal vein once per week at 07,00 before feeding from -8 weeks before parturition to +4 weeks postpartum. All blood samples were collected into 10-mL vacutainer tubes (Becton Dickinson, Franklin Lakes, NJ, USA) and allowed to clot and kept at 4 °C until separation of serum. Clotted blood was centrifuged at 2090 × g at 4 °C for 20 min (Rotanta 460 R centrifuge, Hettich Zentrifugan, Tuttlingen, Germany). The separated serum was aspirated from the supernatant gradually by transfer pipets (Fisher Scientific, Toronto, ON, Canada) without disturbing the sediment. The separated serum was transferred to a sterile 10-mL plastic test tube (Fisher Scientific, Toronto, ON, Canada). All serum samples were stored at -80 °C until analysis to avoid loss of bioactivity and prevent contamination and were thawed on ice for approximately 2 h before use.

Cows were milked twice per day at 05.00 and 16.00 h, and milk samples collected on d 0, 14, 21, 35, and 49 relative to parturition (day 0 means the day of calving), were used for analysis of milk composition including CP, milk fat, lactose, SCC, MUN, and TS.

### Sample analyses

Serum cytokines: Concentration of IL-1 in the serum was assayed by a commercially available bovine ELISA kit (Cusabio Biotech Co. Ltd., Wuhan, China) with mAb (monoclonal antibodies) specific for IL-1 coated on the walls of the microplate strips provided. The procedure involves a competitive inhibition enzyme immunoassay between biotin-conjugated IL-1 and IL-1 with the pre-coated antibody. All samples (50 μL) were tested in duplicate in microtitration wells with biotin-conjugated IL-1 according to the manufacturer’s instructions. The plates were washed with wash buffer after the incubation for 60 min at 37 °C, followed by addition of 50 μL of horseradish peroxidase (HRP)-avidin. Samples were incubated for 30 min at 37 °C. Then, they were washed 3 times with buffer, and 50 μL substrate A and 50 μL of substrate B reagent were added to each well. After incubation at 37 °C for 15 min, the resulting color reaction was read at 450 nm by a microplate reader (Spectramax 190, Molecular Devices Corporation, Sunnyvale, CA, USA) within 10 min, and the final IL-1 concentration was calculated using a 4-parameter logistic curve fit. The sensitivity of this assay was 250 pg/ml, and the intra-assay CV was < 10 %.

Concentration of IL-6 in the serum was measured with a bovine ELISA kit provided by Uscnk Life Science Inc. (Wuhan, China) as described by the manufacturer. The detection limit of the assay was 7.8 pg/ml and the intra-assay variation of all IL-6 assays was controlled by CV limits of < 10 %. The principle of the IL-6 test involves a sandwich enzyme immunoassay, which exhibits a yellow color change proportional to IL-6 concentration. Samples or standards were added to the microtiter plate wells with a biotin-conjugated antibody specific for IL-6 with all samples in duplicate. Then, HRP-avidin was added and incubated. After 3, 3, 5, 5-tetramethylbenzidine (TMB) substrate and sulphuric acid solution were added, the color change was measured spectrophotometrically at a wavelength of 450 nm (Spectramax 190, Molecular Devices Corporation, Sunnyvale, CA, USA).

Concentration of TNF in the serum was determined by a commercially available bovine ELISA kit (Bethyl Laboratories, Inc., Montgomery, TX, USA) using a method described previously by Iqbal et al. [27]. Briefly, all samples were tested in duplicate and the optical density values were read at 450 nm on a microplate spectrophotometer (Spectramax 190, Molecular Devices Corporation, Sunnyvale, CA, USA). The detection range of TNF assays was between 0.078 and 5 ng/mL, and the intra-assay CV was lower than 10 %.

Serum APP: Methods used for measurement of concentrations of Hp (Tridelta Development Ltd., Co.Kildare, Ireland) and SAA (Tridelta Development Ltd.) in the serum were described previously in detail by Iqbal et al. [28]. In brief, serum samples for SAA analyses were initially diluted 1:500. Samples for Hp were not diluted. The minimum detection limits for Hp and SAA assays were 2.5 mg/mL, and 18.8 ng/mL, respectively. All samples were tested in duplicate and the optical densities were measured at 600 nm for Hp and 450 nm for SAA. The intra-assay variations of the two APP assays was controlled by the CV limits of no more than 10 % and for those greater than 10 % samples were reanalyzed.

Serum metabolites: Quantitative determination of serum lactate, BHBA, and NEFA were measured by an enzymatic colorimetric method using commercially available kits provided by Stanbio Laboratory (Boerne, TX, USA) and Wako Chemicals (Richmond, VA, USA), respectively. The detailed methods have been described previously by Ametaj et al. [29]. Briefly, according to the manufacturer’s instructions, the lower detection limits of the kits were 0.06 mg/dl, 0.125 μmol/l, and 0.50 μEq/l for lactate, BHBA, and NEFA, respectively. The principle of the lactate assay involves reduction in the colorless tetrazolium salt by an NADH-coupled enzymatic reaction to formazan, which develops a red color change proportional to the lactate concentration. BHBA test involves the basic principle of conversion of serum BHBA to acetoacetate and NADH by BHBA dehydrogenase in presence of NAD. Then, the NADH reacts with 2-p-iodophenyl-3-p-nitrophenyl-5-phenyltetrazolium chloride (INT) in the presence of diaphorase to form a pink colored adduct proportional to the concentration of BHBA in the serum. The principle of NEFA kit involves acylation of coenzyme A (CoA) by fatty acids in the serum in presence of acyl-CoA synthetase and production of hydrogen peroxide in presence of acyl-CoA oxidase. Hydrogen peroxide, together with peroxidase, permits the oxidative condensation of 3-methyl-N-ethyl-N-β-hydroxy ethyl-O-aniline with 4-aminoantipyrine to produce a purple color change, which is proportional to the serum NEFA concentrations. All samples were tested in duplicate and absorbance of standards and samples vs a blank for lactate, BHBA, and NEFA were read at 492, 505, and 550 nm, respectively, in a microplate reader (Spectramax 190, Molecular Devices Corporation, Sunnyvale, CA, USA). The intra-assay variation of all the three assays was controlled by CV limits of < 10 %.

### Statistical analyses

To perform a standard cross-sectional 2-group study, we compared healthy cows group and SCM cows group at each time point (-8, -4, disease diagnosis, and +4 weeks).

For parametric analysis of the data ANOVA was used by MIXED procedure of SAS (SAS Institute Inc., Cary, NC, USA, Version 9.2) according to the following model:

$$ {\mathrm{Y}}_{\mathrm{i}\mathrm{j}\mathrm{k}} = \upmu +{\mathrm{S}}_{\mathrm{i}}+{\mathrm{W}}_{\mathrm{j}} + {\left(\mathrm{S}\mathrm{W}\right)}_{\mathrm{i}\mathrm{j}} + {\mathrm{e}}_{\mathrm{i}\mathrm{j}\mathrm{k}} $$

where Y_ijk_ is the observations for dependent variables, μ represents the population mean, S_i_ is the fixed effect of health status i (i = 1–2, sick cows compared with healthy control separately), W_j_ is the fixed effect of measurement week j (j = 1–4 or 1–13), SW_ij_ is the fixed effect of health status by week interaction, and e_ijk_ is the residual error.

Measurements taken at different weeks on the same cow were considered as repeated measures in the ANOVA. The variance–covariance structure of the repeated measures was modeled separately for each response variable according to the lowest values of the fit statistics based on the BIC (Bayesian information criteria), and an appropriate structure was fitted. Degrees of freedom were approximated by the method of Kenward-Roger (ddfm = kr).

In order to identify early indicators of SCM, average serum concentrations in the week of diagnosis, -8 and -4 weeks before the expected day of parturition were compared using *t*-test of SAS 9.2 between healthy controls and cows with SCM. Data are exhibited as least-squares means (LSM) and the respective standard error of the mean (SEM). All statistical tests were two-sided. Significance was declared at *P* < 0.05, and tendency was defined at 0.05 < *P* < 0.10.

Multivariate analysis was performed using MetaboAnalyst [30]. Recommended statistical procedures for principal component analysis (PCA) and partial least squares discriminant analysis (PLS-DA) were followed according to previously published protocols [30]. To perform a standard cross-sectional 2-group study, we compared healthy cows group and SCM cows group at each time point (-8, -4, disease diagnosis, and +4 weeks). In the PLS-DA model, a variable importance in the projection (VIP) plot was used to rank the variables based on their importance in discriminating ketosis group from the CON group of cows. Variables with the highest VIP values are the most powerful group discriminators. Typically, VIP values > 1 are significant and VIP values > 2 are highly significant.

Biomarker profiles and the quality of the biomarker sets were determined using receiver-operator characteristic (ROC) curves as calculated by MetaboAnalyst 3.0 [31]. Paired sensitivity and false-positive ratios (1-specificity) at different classification decision boundaries were calculated. A ROC curve is plotted with sensitivity values on the Y-axis and the corresponding false-positive rates (1-specificity) on the X-axis. ROC curves are often summarized into a single metric known as the area under the curve (AUC), which indicates the accuracy of a test for correctly distinguishing one group such as SCM cows from CON ones. If all positive samples are ranked before negative ones, the AUC is 1.0, which indicates a perfectly discriminating test. The 95 % confidence interval (CI) and *P* values were calculated. A rough guide for assessing the utility of a biomarker set based on its AUC is 0.9 ~ 1.0 = excellent; 0.8 ~ 0.9 = good; 0.7 ~ 0.8 = fair; 0.6 ~ 0.7 = poor; 0.5 ~ 0.6 = fail.

## Results

### Serum cytokines

**Fig. 1:**
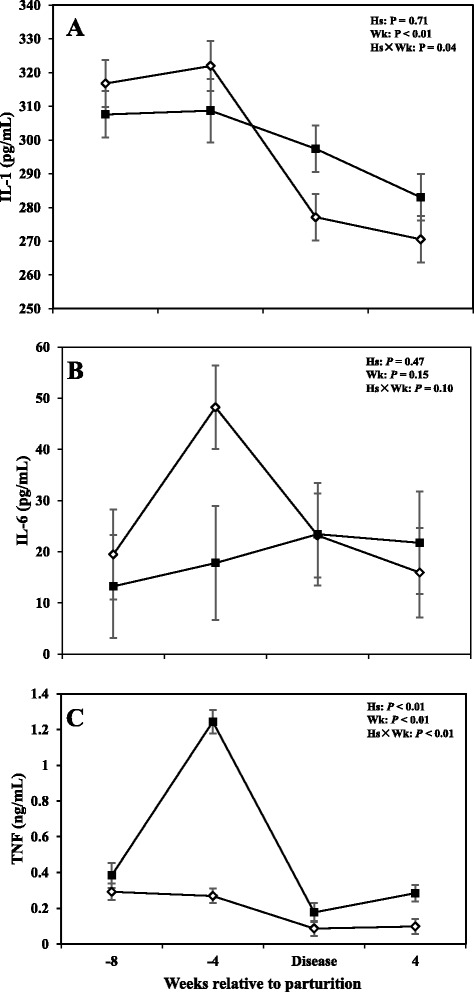
Concentrations of (**a**) interleukin (IL) - 1, (**b**) interleukin (IL) – 6, and (**c**) tumor necrosis factor (TNF) in the serum of periparturient dairy cows with (■, *n* = 6) or without (◇; *n* = 6) subclinical mastitis (SCM) (LSM ± SEM; Hs = effect of health status; Wk = effect of sampling week; Hs × Wk = effect of health status by sampling week interaction)

Data with respects to concentrations of IL-1, IL-6, and TNF in the serum are shown in Fig. 1. Overall data showed that cows with SCM had greater concentrations of TNF throughout the study (*P* < 0.05); with sampling week also having an effect on the results (*P* < 0.0). Comparisons also showed greater concentrations of TNF in cows with SCM at -4 weeks prior to parturition and at the week of diagnosis of the disease (*P* < 0.05) and it continued at +4 week after parturition (0.28 ± 0.04 vs 0.07 ± 0.04; *P* < 0.01). Interleukin-1 was lower in cows with SCM at -4 weeks before parturition (*P* < 0.01) and increased during the week of diagnosis of disease in SCM cows (*P* < 0.05), whereas IL-6 did not show differences between the two groups, during the time points investigated in this study (*P* > 0.05).

### Serum acute phase proteins

**Fig. 2:**
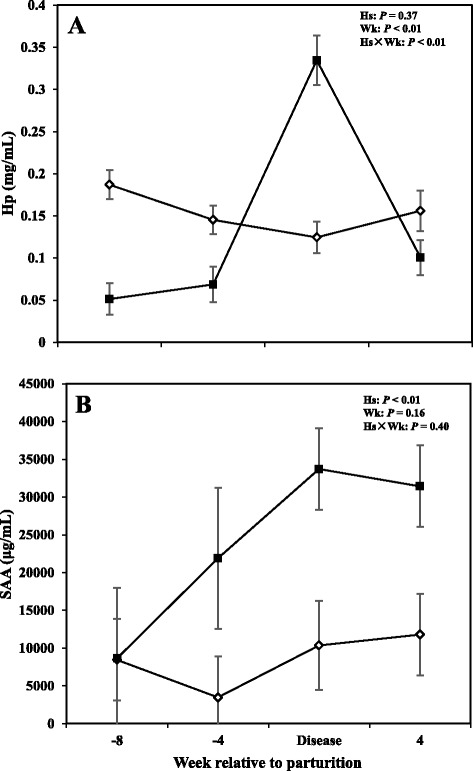
Concentrations of (**a**) haptoglobin (Hp), and (**b**) serum amyloid A (SAA) in the serum of periparturient dairy cows with (■, *n* = 6) or without (◇; *n* = 6) subclinical mastitis (SCM) (LSM ± SEM; Hs = effect of health status; Wk = effect of sampling week; Hs × Wk = effect of health status by sampling week interaction)

Results with regards to concentrations of Hp and SAA in the serum are shown in Fig. 2. Overall data showed no differences in concentrations of Hp between CON cows and those with SCM (*P* > 0.05); with the sampling week and interaction of health status by sampling week having a significant effect (*P* < 0.05). Cows with SCM had lower Hp compared to CON ones at -8 and -4 weeks prior parturition and at +4 weeks after parturition. At the week of diagnosis of disease, concentrations of Hp were greater in cows with SCM (*P* < 0.05).

Overall cows with SCM had greater concentrations of SAA compared to healthy cows throughout the study (*P* < 0.05). Comparison of means at -8 and -4 weeks before parturition did not show significant changes between the two groups. However, results showed a tendency for greater concentrations of SAA (*P* = 0.05) at the week of diagnosis of disease and +4 weeks (*P* = 0.05).

### Serum metabolites

**Fig. 3:**
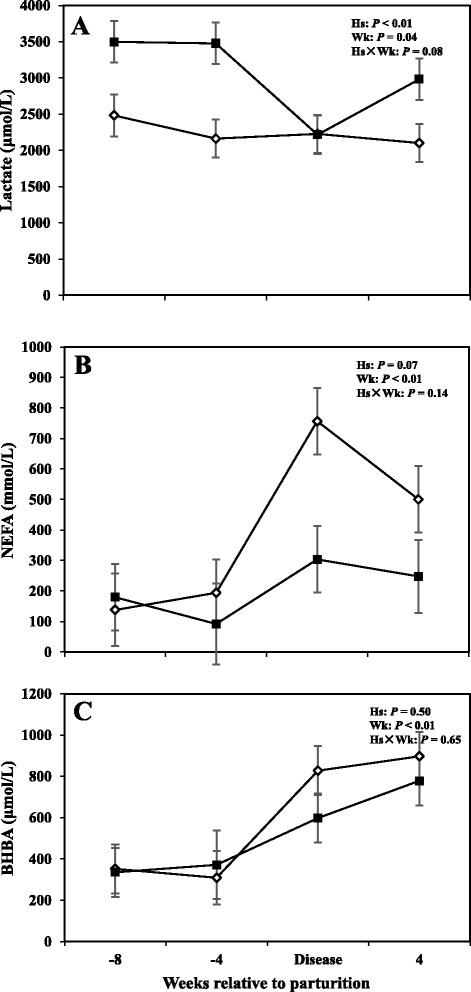
Concentrations of (**a**) lactate, (**b**) non-esterified fatty acids (NEFA), and (**c**) β-hydroxybutyric acid (BHBA) in the serum of periparturient dairy cows with (■, *n* = 6) or without (◇; *n* = 6) subclinical mastitis (SCM) (LSM ± SEM; Hs = effect of health status; Wk = effect of sampling week; Hs × Wk = effect of health status by sampling week interaction)

Alterations in serum metabolites including lactate, BHBA, and NEFA are shown in Fig. 3, whereas concentrations of metabolites for the prepartum period are shown in Tables 1 and 2. Overall data demonstrated that concentrations of lactate in cows with SCM were greater compared to healthy animals (3043 μmol/L vs 2242; *P* < 0.05). The effect of sampling week was also significant (*P* < 0.05), indicating a week-to-week variation in lactate concentration. Furthermore comparison of means at -8 and -4 weeks before parturition showed that cows with SCM had greater concentrations of lactate compared to CON cows (*P* < 0.05). No differences in lactate concentrations were detected between healthy cows and those with SCM at the diagnosis week; however at +4 weeks lactate concentration increased in cows with SCM (2100 ± 129 μmol/L vs 2999 ± 76 μmol/L; *P* < 0.01). Moreover, no differences between the two groups of cows were detected with respect to concentrations of NEFA and BHBA in the serum; however, results showed an effect of week of sampling on both those variables (*P* < 0.05). A tendency for greater concentrations of NEFA (*P* = 0.09) and BHBA (*P* = 0.08) in cows with SCM, at -4 weeks prior to parturition, was observed. Concentrations of lactate and BHBA also were not different between the CON and sick cows during the week when SCM was diagnosed.

**Table 1:** Data of DMI, milk production, milk composition and metabolites, cytokines, and APP in the serum of dairy cows with (*n* = 6) and without subclinical mastitis (SCM) during the periparturient period

	Group^a^			Effect,^b^ *P*-value		
Item	SCM	CON	SEM	Hs	Wk	Hs × Wk
DMI^c^ (kg/d)	16.90	18.64	0.72	0.02	<0.01	0.09
Milk production^d^ (kg/d)	33.76	42.16	2.52	0.04	<0.01	0.37
Milk composition^e^ (g/kg, unless otherwise stated)						
Fat	3.67	3.84	0.23	0.63	0.24	0.23
Protein	2.92	2.86	0.05	0.39	0.04	0.19
Fat:protein ratio	1.27	1.37	0.10	0.51	0.24	0.23
Lactose	4.59	4.56	0.05	0.72	0.03	0.86
SCC (10^3^ cells/mL)	628.44	29.38	124.45	<0.01	0.20	0.21
Milk urea N (mg/dL)	17.43	15.61	1.02	0.24	0.53	0.38
TS	12.21	12.21	0.20	0.98	0.40	0.44
Serum parameters^f^						
Lactate (μmol/L)	3,043.38	2,242.94	149.29	<0.01	0.04	0.08
NEFA (mmol/L)	205.46	397.17	64.62	0.07	<0.01	0.14
BHBA (μmol/L)	520.81	596.03	73.09	0.50	<0.01	0.65
IL-1 (pg/mL)	299.19	296.62	4.72	0.71	<0.01	0.04
IL-6 (pg/mL)	19.05	26.70	6.42	0.47	0.15	0.10
TNF (ng/mL)	0.52	0.19	0.04	<0.01	<0.01	<0.01
Haptoglobin (mg/mL)	0.14	0.15	0.01	0.37	<0.01	<0.01
SAA (ug/mL)	23,915	8,514.32	2,517.79	<0.01	0.16	0.40

^a^CON = cows without subclinical mastitis (health control); SCM = cows with subclinical mastitis

^b^Effect of health status (Hs), sampling week (Wk), and health status by sampling week interaction (Hs × Wk)

^c^Dry matter intake was calculated from week -8 to +8 relative to parturition

^d^Milk production was calculated from week +1 to +8 relative to parturition

^e^Milk compositions were determined on week +2, +3, +5, +7 relative to parturition

^f^Serum parameters were calculated from week -8, -4, disease, and +4 relative to parturition

**Table 2:** Data of DMI, milk production, milk composition, and various serum variables related to carbohydrate metabolism as well as to proinflammatory cytokines and acute phase proteins at the diagnosis week, and prior to the diagnosis of subclinical mastitis (SCM) at -8 and -4 weeks prior to expected day of parturition

	-8 weeks before parturation			4 weeks before parturation			SCM diagnosis week^a^			4 weeks after parturition		
Item	CON	SCM	*P*-value	CON	SCM	*P*-value	CON	SCM	*P*-value	CON P-value	SCM	*P*-value
DMI (kg/d)	16.27 ± 1.04	14.94 ± 1.04	0.42	15.93 ± 0.10	12.98 ± 0.75	0.05	20.34 ± 0.56	16.23 ± 0.44	<0.01	21.16 ± 0.68	22.46 ± 1.79	0.23
Milk production (kg/d)							43.01 ± 1.62	28.23 ± 3.95	<0.01	42.04 ± 2.21	35.36 ± 3.96	0.17
Milk composition (g/kg, unless otherwise stated)												
Fat							5.08 ± 0.45	3.32 ± 0.41	0.02			
Protein							3.00 ± 0.10	2.97 ± 0.09	0.86			
Fat : Protein ratio							1.69 ± 0.12	1.11 ± 0.12	<0.01			
Lactose							4.54 ± 0.05	4.53 ± 0.06	0.92			
SCC (10^3^ cells/mL)							28.33 ± 5.63	1,867 ± 699	0.02			
Milk urea N (mg/dL)							15.39 ± 0.76	18.70 ± 1.11	0.03			
TS							12.21 ± 0.31	12.19 ± 0.26	0.95			
Serum parameters												
Lactate (μmol/L)	2,455 ± 349	3,478 ± 153	0.03	2,162 ± 184	3,467 ± 546	0.04	2,228 ± 320	2,215 ± 203	0.98	2,100 ± 129	2,999 ± 76	<0.01
NEFA (mmol/L)	141 ± 32.77	179 ± 42.91	0.51	194 ± 47.17	92.97 ± 10.42	0.09	756 ± 232	303 ± 55	0.11	500.43 ± 151.71	243.89 ± 39.28	0.16
BHBA (μmol/L)	352 ± 37.71	336 ± 42.62	0.78	312 ± 18.50	354 ± 3.32	0.08	827 ± 151	598 ± 176	0.35	896.73 ± 188.3	777.8 ± 91.43	0.58
IL-1 (pg/mL)	316.79 ± 6.04	308 ± 13.76	0.55	321 ± 1.59	306. ± 1.46	<0.01	277. ± 5.42	297 ± 4.50	0.02	270.57 ± 2.87	283.02 ± 7.22	0.14
IL-6 (pg/mL)	19.23 ± 5.67	13.23 ± 3.15	0.42	48.24 ± 17.51	14.75 ± 6.13	0.24	23.17 ± 5.18	23.43 ± 4.89	0.97	15.71 ± 3.27	21.73 ± 6.02	0.38
TNF (ng/mL)	0.34 ± 0.03	0.35 ± 0.02	0.96	0.27 ± 0.05	1.29 ± 0.05	<0.01	0.06 ± 0.03	0.23 ± 0.01	0.01	0.07 ± 0.04	0.28 ± 0.04	0.01
Haptoglobin (mg/mL)	0.19 ± 0.03	0.05 ± 0.01	<0.01	0.15 ± 0.01	0.07 ± 0.01	<0.01	0.12 ± 0.01	0.33 ± 0.02	<0.01	0.16 ± 0.01	0.10 ± 0.02	0.04
SAA (ug/mL)	8,448 ± 3,373	8,799 ± 1,464	0.96	3,461 ± 342	21,052 ± 12,162	0.39	10,401 ± 1722	33,705 ± 9,459	0.05	11,797 ± 1,853	31,419 ± 8,039	0.05

^a^Cows were diagnosed with subclinical mastitis (*n* = 6) ranging from week +2 to +3. CON = cows without subclinical mastitis (health control); SCM = cows with subclinical mastitis

### Dry matter intake, milk production, and milk composition

**Fig. 4:**
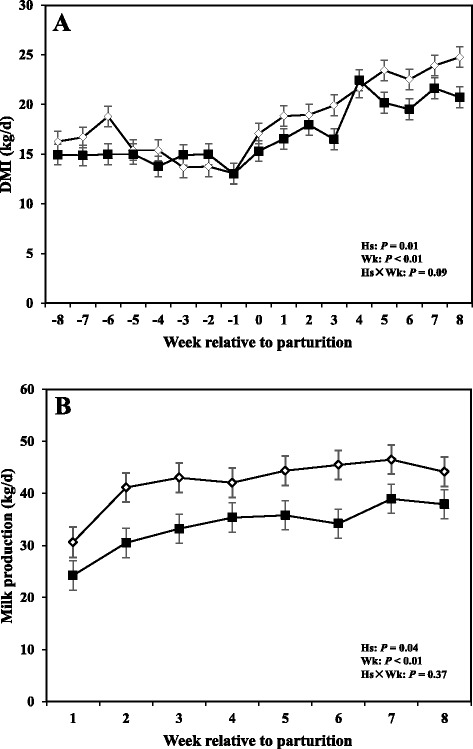
**a** DMI (Dry Matter intake), and (**b**) milk production of periparturient dairy cows with (■, *n* = 6) or without (◇; *n* = 6) subclinical mastitis (SCM) (LSM ± SEM; Hs = effect of health status; Wk = effect of sampling week; Hs × Wk = effect of health status by sampling week interaction)

**Fig. 5:**
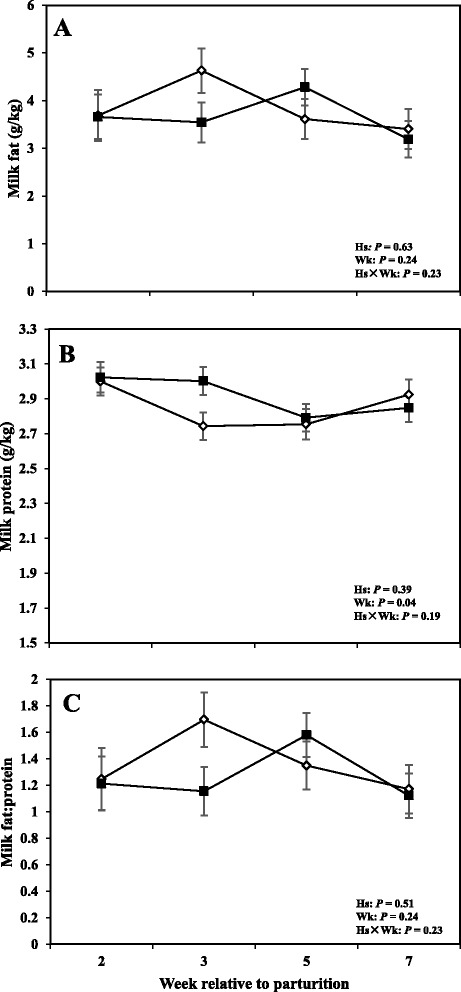
Concentrations of (**a**) fat, (**b**) protein, and (**c**) fat:protein ratio in the milk of periparturient dairy cows with (■, *n* = 6) or without (◇; *n* = 6) subclinical mastitis (SCM) (LSM ± SEM; Hs = effect of health status; Wk = effect of sampling week; Hs × Wk = effect of health status by sampling week interaction)

**Fig. 6:**
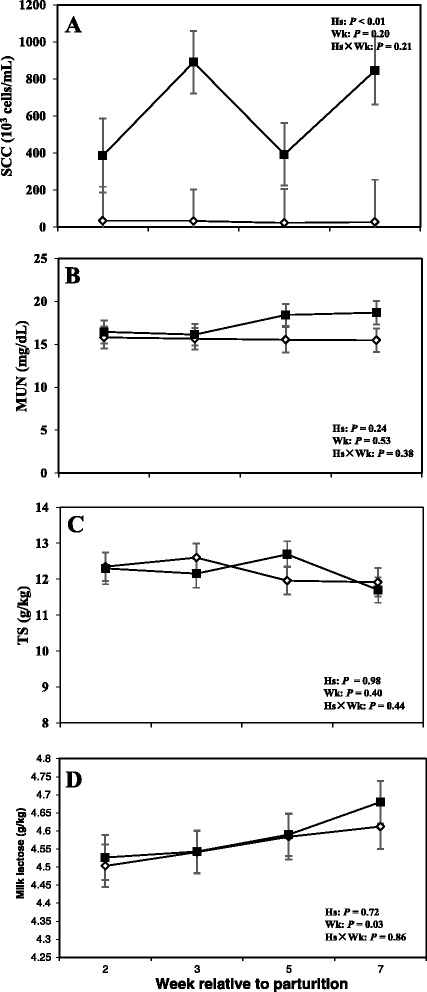
Concentrations of (**a**) somatic cell count (SCC), (**b**) milk urea N (MUN), (**c**) total solid (TS), and (**d**) lactose in the milk of periparturient dairy cows with (■, *n* = 6) or without (◇; *n* = 6) subclinical mastitis (SCM) (LSM ± SEM; Hs = effect of health status; Wk = effect of sampling week; Hs × Wk = effect of health status by sampling week interaction

Data related to DMI and milk production are shown in Fig. 4, whereas concentrations of milk fat, protein, and fat:protein ratio in the milk are presented in Fig. 5. Moreover SCC, TS, and lactose in the milk are shown in Fig. 6.

Overall cows with SCM had lower DMI intake compared to healthy cows (*P* < 0.05). The sampling week had an effect (*P* < 0.05), indicating a week-to-week variation in DMI. Comparison of means demonstrated that DMI tended to be lower at -4 weeks before parturition (*P* = 0.05) and was lower in cows with SCM during the week of diagnosis of disease (*P* < 0.05).

Moreover cows with SCM showed lower milk production throughout the postpartum period and especially during the week of diagnosis of disease (28.23 ± 3.95 vs 43.01 ± 1.62; *P* < 0.01). Fat content was not different between the two groups; however, at the week of diagnosis of SCM, those cows had significantly lower milk fat content (3.32 ± 0.41 vs 5.08 ± 0.45; *P* < 0.05). Moreover, fat to protein ratio in the milk was lower in SCM cows compared to healthy cows at the week of SCM diagnosis (*P* < 0.05). In addition, SCC were greater in cows with SCM compared to healthy animals throughout the postpartal period and during the week of SCM diagnosis (1867 ± 698.95 vs 28.33 ± 5.63; *P* < 0.05). Cows with SCM also had greater MUN at the week of diagnosis of SCM, whereas lactose and TS did not show differences between the two groups (Table 2).

### Multivariate analysis

**Fig. 7:**
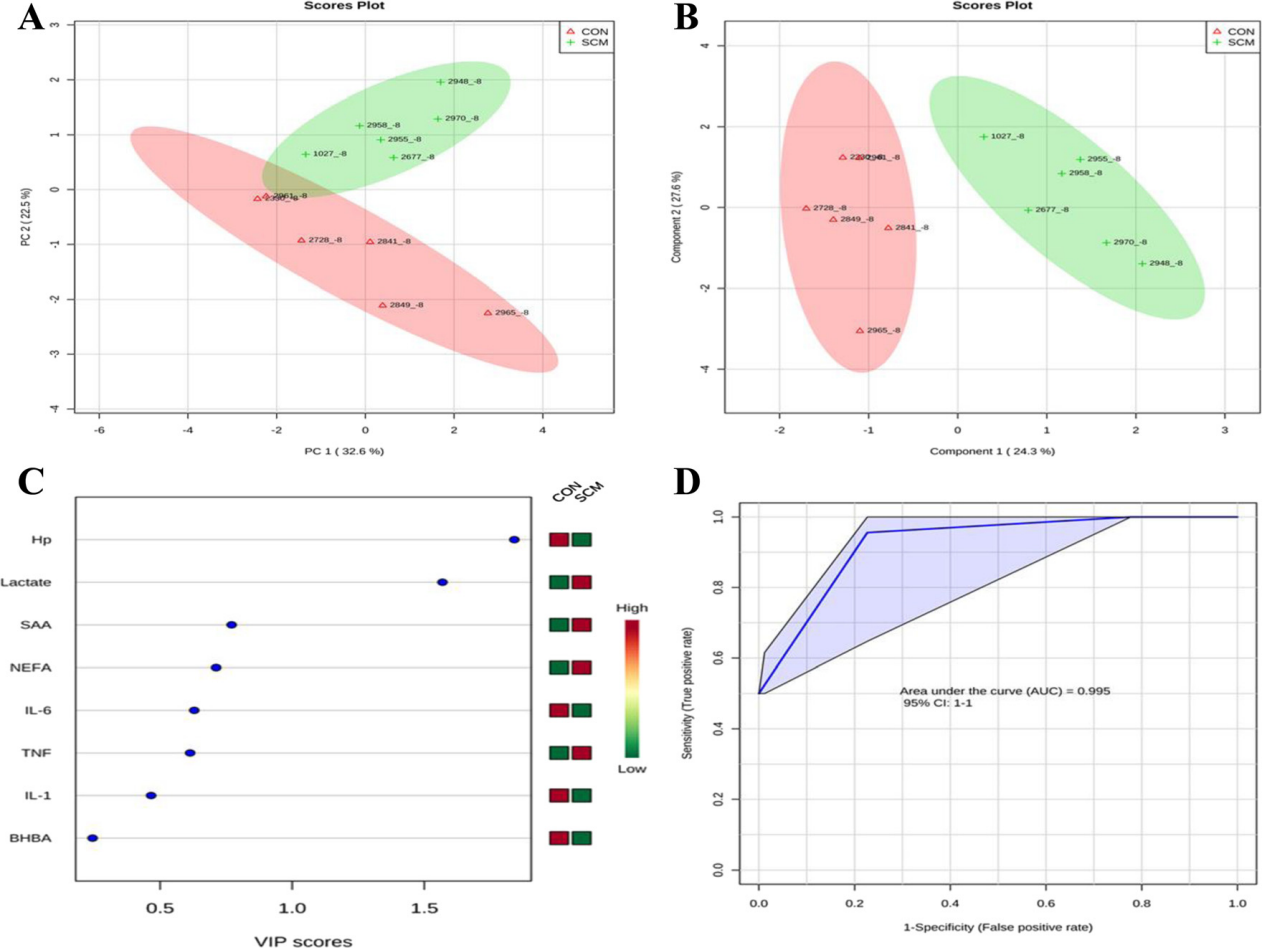
**a** Principal component analysis (PCA) and (**b**) Partial least squares-discriminant analysis (PLS-DA) of 6 control (red triangles), and 6 subclinical mastitis cows (green + sign), at -8 weeks before parturition showing 2 separated clusters for 2 groups. **c** Variables ranked by variable importance in projection (VIP), and (**d**) Receiver-operator characteristic (ROC) curve of 6 CON and 6 SCM cows at -8 weeks before parturition for the top 3 serum variables

**Fig. 8:**
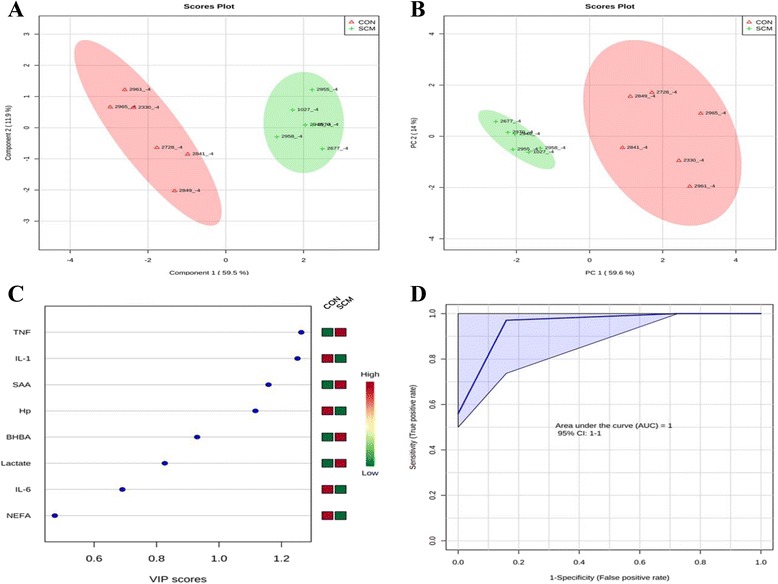
**a** PCA and (**b**) PLS-DA of 6 controls (red triangles), and 6 subclinical mastitis cows (green + sign) at -4 week before parturition showing 2 separated clusters for 2 groups. **c** VIP, and (**d**) ROC curve of 6 CON and 6 SCM cows at -4 weeks before parturition for the top 3 serum variables

**Fig. 9:**
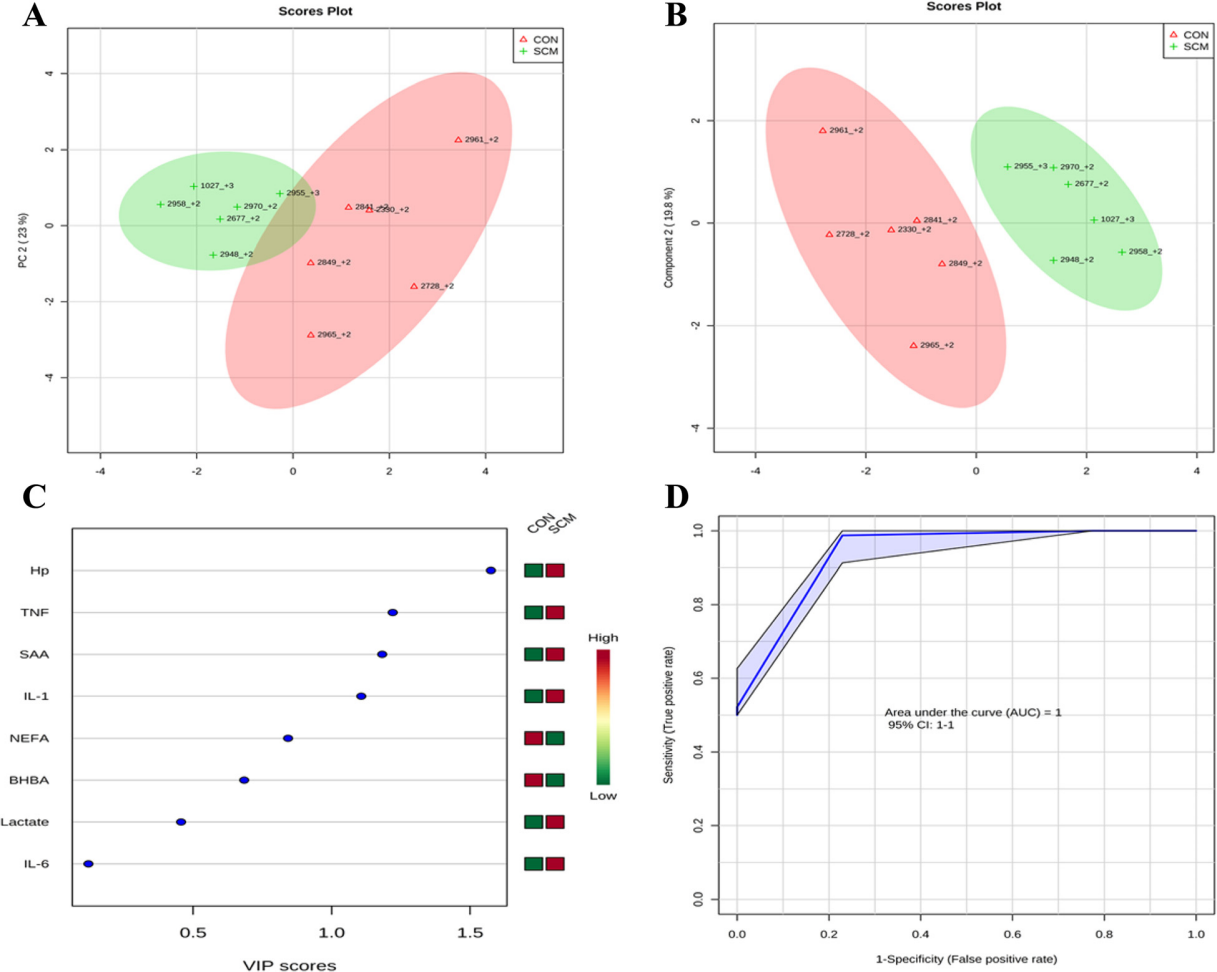
**a** PCA and (**b**) PLS-DA of 6 controls (red triangles), and 6 subclinical mastitis cows (green + sign), at disease week showing 2 separated clusters for 2 groups. **c** VIP, and (**d**) ROC curve of 6 CON and 6 SCM cows at disease week for the top 3 serum variables

**Fig. 10:**
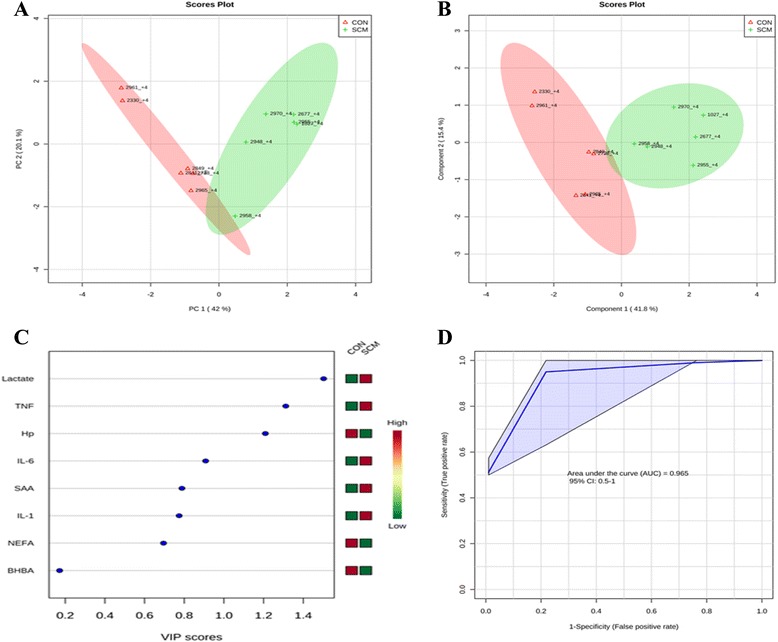
**a** PCA and (**b**) PLS-DA of 6 controls (red triangles), and 6 subclinical mastitis (green + sign), at 4 week after parturition showing 2 separated clusters for 2 groups. **c** VIP, and (**d**) ROC curve of 6 CON and 6 SCM cows at +4 weeks after parturition for the top 3 serum variables

Comparisons between the healthy cows and SM ones at -8 weeks prepartum, PCA (Principal Componenet Analysis) analysis showed that the first 2 PC (principal components) covered 55.9 % of the observed variance in the sample set (Fig. 7a). In addition PLS-DA scores plot revealed that it is possible to discriminate between healthy cows and those with SCM starting at -8 weeks prior to the expected day of parturition (Fig. 7b). When healthy cows and SCM ones were compared at -4 week prior to parturition (Fig. 8a), PCA analysis showed that the first 2 PC covered 75.9 % of the observed variance in the sample. PLS-DA scores plot also revealed that it is possible to discriminate between CON cows and those with SCM starting at -4 weeks before the expected day of parturition (Fig. 8b). Similar results were obtained for the week of diagnosis of SCM (Fig. 9a and b). A VIP plot in a PLS-DA model at 4 time points in which the serum variables were ranked based on their contribution to discriminating the SM cows from CON ones are shown in Figs. 7c, 8c, 9c and 10c. The VIP plots indicated that Hp lactate, and SAA at -8 weeks; TNF, IL-1, and SAA at -4 weeks; Hp, TNF and SAA at the week of diagnosis of SCM; and lactate, TNF, and Hp at +4 weeks were the strongest discriminating variables for separating SCM cases form CON cows. The heat map on the right side of the 4 VIP plots indicated that these variables were enhanced in cows with SCM relative to CON cows.

A ROC curve plot indicating the performance of the top 3 metabolites in predicting which cows will develop SCM at -8 and -4 weeks using a PLS-DA model are shown in Figs. 7d and 8d. The AUC for the curve at -8 weeks is 0.995 (95 % CI, 1-1), which indicates that Hp lactate, and SAA together, at -8 weeks have strong predictive abilities. The AUC for the curve at -4 weeks is 1 (95 % CI, 1-1), which suggest that at – 4 weeks the combination of TNF and IL-1 have very strong predictive abilities. These results demonstrate that biomarker models developed at -8 and -4 weeks could be used to predict which cows are susceptible to develop SCM after parturition. The week of diagnosis of disease was at +2 to +3 weeks postpartum. The ROC curve developed based on three metabolites Hp, TNF and SAA at the week of diagnosis of SCM indicates that this three-metabolite set were a highly significant biomarker for diagnosis of SCM: AUC, 1 (95 % CI, 1-1, Fig. 9d). Moreover, multivariate models (ROC curves) combining 3 discriminating variables (i.e., lactate, TNF, and Hp) at +4 weeks produced an area under the receiver-operating curve of 0.965 (95 % CI: 0.5–1, Fig. 10d).

## Discussion

We hypothesized that alterations of innate immunity reactants as well as changes in carbohydrate and lipid metabolism could precede development of SCM in transition dairy cows. Indeed, results revealed multiple alterations in innate immunity and metabolic variables several weeks prior to the expected day of parturition and prior to diagnosis of SCM. Cows affected by SCM had greater concentration of TNF, SAA, and lactate, lower DMI and milk production, and an increase in milk SCC.

Concentrations of TNF were greater in the serum of cows with SCM at -4 weeks before the expected day of parturition and at the week of SCM diagnosis, compared to their healthy counterparts. Tumor necrosis factor has been related previously to inflammation of the udder [32]. In LPS-induced mastitis models, TNF is detected in the plasma and milk of early lactating cows [33, 34]. In addition, during natural coliform mastitis or experimental *E. coli* infection TNF is significantly increased in both milk and serum [34, 35]. However, it should be noted that in serum, concentration of TNF is only extensively elevated in severe clinical cases of coliform mastitis [34, 36]. Results of the study also showed that serum IL-1 was greater in SCM cows at the week of SCM diagnosis but lower at -4 weeks prior to parturition. Greater concentrations of IL-1 and TNF in the serum, at the week of SCM diagnosis, suggest presence of inflammation in the udder. TNF and IL-1 are produced by activated macrophages and neutrophils present in the infected mammary gland [37]. Concentrations of IL-1 and TNF in the milk are related to recruitment of neutrophils to the mammary gland [33]. Both IL-1 and TNF serve as activators of APP production in liver hepatocytes [33, 38]. To our best knowledge this is the first study to report that SCM was preceded by elevation of TNF concentrations starting at -4 weeks before the expected day of parturition, suggesting that TNF might be used as early screening biomarker of disease state in transition dairy cows. It has been previously reported that E. coli or LPS-induced mastitis induced a quick and strong transcriptome response in liver, causing up- regulation of acute phase proteins genes [39, 40].

Circulatory pro-inflammatory cytokines are known to trigger production of APP from liver hepatic cells, increasing concentrations of peripheral plasma APP around parturition [16, 41]. Indeed TNF concentrations and serum SAA were greater in SCM cows. However, results of this study showed lower concentrations of Hp in SCM cows versus healthy cows at -8 and -4 weeks prepartum. Since cows affected by SCM were in an inflammatory state during the dry off, as indicated by greater concentrations of TNF and SAA, it is possible that lower Hp in their blood during -8 and -4 weeks prior to parturition might suggest movement of Hp into the udder to help with immune responses. Haptoglobin actively participates in all the inflammatory processes from neutrophil recruitment and free radical quenching, to tissue repair and regeneration [42]. On another note, lowering of Hp protein in the blood can make cows susceptible to infection and inflammatory diseases during the transition period. It should be pointed out that plasma concentrations of Hp increased in SCM cows during the week of diagnosis of SCM. These results are in line with those of Humblet et al. [43] and Rezamand et al. [44]. Haptoglobin scavenges hemoglobin and prevents utilization of iron by bacteria translocated into the blood systemic circulation [45], and plasma Hp is considered an indicator of inflammation and infection [43].

In our study concentrations of SAA in the serum were greater in cows with SCM throughout the study. Acute phase proteins have been reported to increase in serum and milk of cows with clinical and subclinical mastitis. Our results are in agreement with other authors that have reported about SAA and mastitis [23, 46, 47]. Serum amyloid A binds to high-density lipoproteins and participates in expedited clearance of translocated endotoxin through the liver [48]. In addition, SAA is present in secretory epithelial cells of the mammary gland at significantly greater levels in infected udders and may play a significant role in early response to invasion of mammary tissues by pathogenic bacteria and might protect the teat potential colonization of that area by bacteria during milking or suckling [16, 23]. Based on greater TNF and SAA during the dry off period it is speculated that the infection of the udder might have started immediately after drying off and cows of SCM group have been in a state of endotoxemia during the entire dry off period and immediately after calving.

One of the most interesting finding of this study was that cows with SCM had greater concentrations of lactate in the serum starting at -8 weeks before parturition. Several authors have described elevation of lactate concentrations in the milk during presence of clinical mastitis, suggesting that lactate could be a good indicator of udder health [12, 13]. Lactate also is an indicator of death in humans and ruminants [49, 50]. It should be kept in mind that SM is diagnosed based on the number of somatic cells in the milk postpartum. Previously, it has been reported that high levels of milk lactate are closely related to SCC [13], making lactate a potential indicator of clinical and subclinical mastitis. High lactate also happens during sepsis or endotoxemia. These data support our assumption SCM cows were in a state of endotoxemia because during these conditions there is enhanced peripheral glucose utilization and increased gluconeogenesis [51]. For example Lang et al. [52] showed that the major gluconeogenic precursor, lactate, was markedly elevated following endotoxin administration. Lactate is also produced from monocytes/macrophages [53]. Moreover proinflammatory cytokines like TNF and IL-1 have been shown to increase glycolysis but lower oxidation of glucose within the tricarboxylic cycle, contributing to elevation of lactate [54]. To our best knowledge, this is the first study to relate concentrations of plasma lactate with SCM starting at -8 weeks prior to the expected day of parturition. Intriguingly lactate inhibits affect T cell motility to the inflammation site and its effector functions [55]. In addition lactate suppresses T cell cytotoxic activity [56], alter antigen-presenting ability of dendritic cells [57], and interfere with NK cell activity [58].

Dry matter intake was lower starting at -4 weeks before parturition and was lower in cows affected by SCM. One of the most important physiological changes occurring during dry off period is a decrease in DMI [59] and lower feed intake is known to be associated with a drop in body weight. Gonzalez et al. [60] reported that changes in eating behavior are potential indicators for monitoring health disorders of dairy cows. In addition, Huzzey et al. [61] showed that prepartum DMI is able to identify cows at risk for metritis. Dry matter intake, and subsequently nutrient intake, is lowered before calving and remains low for a few days after calving. This period often represents a time when the immune system of the cow is severely suppressed, making cows particularly vulnerable to infectious diseases such as mastitis [62]. Endotoxemia lowers DMI [63, 64], therefore it is hypothesized that cows (not yet diseased) in our experiment might have been experiencing endotoxemia at -4 weeks before calving. The reason for the decrease in DMI in SCM cows might be the increase in TNF concentration. Proinflammatory cytokines are known to affect feed intake and in this study TNF was greater in dairy cows prior to diagnosis of SCM [65]. These results suggest that DMI might be an early indicator of SCM in dairy cows.

Subclinical mastitis is associated with lowering of milk production and significant financial losses to the dairy industry [66]. In our study cows with SCM had lower milk production throughout the postpartal experimental period. The reason why cows with SCM had lower milk production can be related to increased concentrations of TNF in the serum. It has been reported that infection by Gram-negative bacteria and their cell wall component, lipopolysaccharide (LPS), trigger inhibition of prolactin production, indirectly through TNF, in the pituitary gland Theas et al. [67]. Prolactin is a known hormone related to stimulation of milk production. This assumption is supported by a previous investigation demonstrating a decrease in milk yield in lactating cows after parenteral administration of TNF Kushibiki et al. [68].

During SCM no visible changes occur in the appearance of the udder; however, milk production decreases, SCC increases, and milk composition is altered [3]. Indeed in the present study SCM was associated with a decrease in milk production and alterations in milk composition. Cows with SCM had greater SCC and MUN, lower milk fat and fat:protein ratio during the week of disease diagnosis (*P* < 0.05). Milk fat is often used as an indicator of rumen health and forage intake [69, 70] and has been often used as an indicator of subacute ruminal acidosis (SARA) in dairy cows. Duffield et al. [71] suggested the ratio of fat: protein as a breakpoint for ketosis in dairy cows. In addition, lower fat:protein ratio in cows with SCM indicates that these cows are at high risk of SARA. The results of our study suggest that SCM cows might have been in a state of endotoxemia. Previously, Zebeli and Ametaj [72] reported a strong correlation between rumen endotoxin and milk fat content. During endotoxemia lipids deflect to liver instead of supporting the udder lipid metabolism because lipoproteins that bind and carry endotoxin diverge to the liver to excrete LPS through bile. This might have contributed to lower fat content in the milk. As expected cows with SCM showed greater SCC in the milk compared with healthy cows (*P* < 0.05) throughout the postpartal period, which served also for diagnosis of cows with SCM. Of note cows going through SCM are at risk of developing clinical mastitis [44, 73]. Reduced MUN, milk protein, and lactose synthesis have been previously described to be lower in cows with SCM, and may indicate a transient dietary protein deficiency [44]. In addition Obled et al. [74] demonstrated that during inflammation and disease states, the requirement for some amino acids are significantly increased.

## Conclusions

Overall, the results of this study indicate that cows affected by SCM display an activated innate immunity and altered carbohydrate metabolism weeks before of diagnosis of the disease. More specifically, cows with SCM had greater serum lactate, SAA, TNF, and lactate prior to parturition and at diagnosis of SCM. In addition subclinically diseased cows showed alterations in milk production, milk composition, and DMI. Furthermore multivariate analysis of the data (PCA and PLS-DA) supports the fact that it is possible to discriminate between healthy cows and those with SCM at -8 weeks before parturition. Results of our study support the idea that Hp lactate, and SAA, at -8 weeks, and TNF and IL-1 at -4 weeks might be used to screen cows during dry off for disease state. Since innate immunity is a general non-specific immune response the identified variables might be used to indicate a non-health status more than a specific disease. More research and a greater number of animals are warranted to understand the pathogenesis of SCM and to validate these results in the future.
